# Evaluating the Survival Benefits of Perioperative Chemotherapy in Frail and Morbid Muscle-Invasive Bladder Cancer Patients †

**DOI:** 10.3390/jpm14090954

**Published:** 2024-09-09

**Authors:** Ziv Savin, Lin Levin, Alon Lazarovich, Barak Rosenzweig, Reut Shashar, Azik Hoffman, Jonathan Gal, Miki Haifler, Ilona Pilosov, Yuval Freifeld, Sagi Arieh Shpitzer, Shay Golan, Roy Mano, Ofer Yossepowitch

**Affiliations:** 1 Department of Urology, Tel-Aviv Sourasky Medical Center, Tel-Aviv University, Tel-Aviv 6423906, Israel; 2 Faculty of Medicine, Hebrew University of Jerusalem, Jerusalem 9190500, Israel; lin.levin@mail.huji.ac.il; 3 Department of Urology, Chaim Sheba Medical Center, Tel-Aviv University, Ramat Gan 5266202, Israelbarak.rosenzweig@sheba.health.gov.il (B.R.); 4 Israeli Urologic Oncology Collaboration (IUOC); yuvalfr@clalit.org.il (Y.F.); shayg@clalit.org.il (S.G.); 5 Department of Urology, Rambam Health Center, Technion Israel Institute of Technology, Haifa 3109601, Israel; 6 Department of Urology, Shamir Medical Center, Tel-Aviv University, Tel Aviv 6997801, Israel; 7 Department of Urology, Meir Medical Center, Tel-Aviv University, Kfar-Saba 4428164, Israel; 8 Department of Urology, Carmel Medical Center, Technion Israel Institute of Technology, Haifa 3436212, Israel; 9 Department of Urology, Rabin Medical Center, Tel-Aviv University, Petach-Tikva 4941492, Israel

**Keywords:** neoadjuvant chemotherapy, adjuvant chemotherapy, frailty, comorbidity index, bladder cancer

## Abstract

Introduction: Current guidelines endorse the use of perioperative chemotherapy (POC) in muscle-invasive bladder cancer (MIBC) to enhance the long-term overall survival (OS) compared to radical cystectomy (RC) alone. This study aims to assess the impact of POC on the OS in frail and morbid (F-M) patients undergoing RC. Methods: A retrospective multicenter study of 291 patients who underwent RC between 2015 and 2019 was performed. Patients with both a Charlson comorbidity index ≥ 4 and Modified Frailty Index ≥ 2 were classified as the F-M cohort. We compared the clinical and pathological characteristics and outcomes of the F-M patients who received POC to those who underwent RC alone. Univariable and multivariable analyses were performed to identify the predictors of the OS. Results: The F-M cohort included 102 patients. POC was administered to 44% of these patients: neoadjuvant (NAC) to 31%, adjuvant (AC) to 19%, and both to 6 (6%). The OS was significantly lower in the F-M cohort compared to in the healthier patients (median OS 42 months, *p* = 0.02). The F-M patients who received POC were younger, less morbid and had better renal function. Although POC was marginally associated with improved OS in the univariable analysis (*p* = 0.06), this was not significant in the multivariable analysis (*p* = 0.50). NAC was associated with improved OS in the univariable analysis (*p* = 0.004) but not after adjustment for competing factors (*p* = 1.00). AC was not associated with the OS. Conclusions: POC does not improve the OS in F-M patients undergoing RC. Personalized treatment strategies and further prospective studies are needed to optimize care in this unique vulnerable population.

## 1. Introduction

Muscle-invasive bladder cancer (MIBC) is an aggressive disease commonly requiring a multimodal treatment approach [1]. While radical cystectomy (RC) is still considered the cornerstone of MIBC treatment, contemporary evidence emphasizes the clinical benefit associated with the administration of perioperative chemotherapy (POC), particularly in the neoadjuvant setting [2]. Neoadjuvant chemotherapy (NAC), considered the standard of care for MIBC, has been demonstrated to improve the long-term overall survival (OS) by 8–10% compared to RC alone [3], whereas adjuvant chemotherapy (AC) is supported by a lower level of evidence. Patients with adverse pathology, namely, locally advanced disease and/or lymph node metastasis (pT3-4, pN+), may benefit from AC [4,5,6]. Despite its unequivocal advantage, the side effect profile and toxicity associated with Cisplatin-based chemotherapy remain a major clinical concern [7].

Frailty, characterized by reduced physiological reserves and comorbidities, is prevalent among elderly patients with MIBC. Given a median age of 73 years at diagnosis of MIBC in the US and a frailty rate affecting up to 21% of urological patients over 70 [8,9], many of the patients diagnosed with MIBC will ultimately be categorized as frail and comorbid. Given the generally low overall survival (OS) and purported increased chemotherapy-related toxicity in this population, the pertinence and efficacy of POC become dubious [10,11]. Several studies have explored the influence of age, comorbidities, and frailty on MIBC treatment [12,13,14], yet the clinical impact of POC in frail and morbid individuals remains elusive. 

The current clinical guidelines addressing the issue of POC in patients with MIBC do not specifically focus on the issue of frailty or morbidity. Hence, we sought to investigate the impact of POC on the OS in frail and morbid patients diagnosed with MIBC. 

## 2. Methods

### 2.1. Study Population

Following institutional review board approval (Helsinki Committee, TLV-0483-23, 14 November 2023), we conducted a retrospective multicenter analysis of 291 consecutive patients who underwent RC for MIBC between 2015 and 2019 across six medical centers. Patient staging was determined using the TNM classification system based on both imaging and histological findings [15,16]. Metastatic patients were excluded from the study. Eligible patients diagnosed with MIBC were treated with curative intent and underwent RC via either open or laparoscopic/robotic approaches. The surgery involved removal of the bladder and pelvic lymph nodes and urinary diversion. In male patients, the procedure also included the removal of the prostate, seminal vesicles, and distal ureters, and in female patients it included the removal of the uterus, ovaries, anterior vaginal wall and distal ureters. The type of urinary diversion was selected based on the patient’s age, baseline clinical and oncological characteristics and personal preference. Dedicated genitourinary pathologists reviewed all RC specimens for histology, TNM staging, surgical margins and lymph node status. Postoperative follow-up, scheduled at 6- to 12-month intervals, included cross-sectional imaging and laboratory workup. Based on the literature, 30–50% of RC patients receive POC, and the 2–3-year mortality rates of elderly frail patients undergoing RC is 30–60% [17,18]. The estimated minimal sample size at a 5% level of significance and 80% power for difference between mortality rates of at least 30% is 84 patients. We set our goal for at least 100 patients for the F-M cohort. 

### 2.2. Frailty and Morbidity Assessment 

In this study, we employed 2 indexes commonly utilized to estimate morbidity and frailty, namely, the Modified Frailty Index (mFI) and the Charlson Comorbidity Index (CCI) [19,20]. The mFI is calculated by 11 preoperative medical conditions, as previously described by Chappidi et al. (Supplementary Table S1) [20]. The CCI described by Koppie and colleagues is determined using 19 preoperative comorbidities with additional points given for age (Supplementary Table S2) [21]. In our study, patients meeting both criteria of mFI ≥ 2 and CCI ≥ 4 were classified into a frail and morbid category (F-M cohort). Preoperative clinical characteristics were compared between the F-M cohort and the non-frail and healthier patients, including age, serum albumin levels and estimated glomerular filtration rate (eGFR) calculated by means of the Modification of Diet in Renal Disease formula [22].

### 2.3. Perioperative Chemotherapy

NAC was offered to Cisplatin-eligible patients based on Galsky criteria, including Eastern Cooperative Oncology Group (ECOG) performance status of 0 to 1, renal function with an estimated glomerular filtration rate (eGFR) above 60 mL/min/1.73 m^2^, and the absence of significant heart failure, hearing loss, or neuropathy [23]. AC was offered postoperatively to Cisplatin-eligible patients with adverse pathology (pT3/4 or lymph nodes invasion). The decision regarding administration of perioperative chemotherapy was made in a multidisciplinary forum at each participating institution after comprehensive discussion with the patient considering oncological outcomes, toxicity and side effects, compliance and personal preference. In some patients with adverse pathology at RC following NAC, a decision was rendered to continue with AC after surgery. Standard Cisplatin-based regimes including Methotrexate, Vinblastine, Adriamycin and Cisplatin (MVAC), Dense-dose MVAC and Gemcitabine-Cisplatin (GC), were administrated. Neoadjuvant or adjuvant immunotherapy was not offered to any of the patients during the study years. The F-M cohort was further stratified according to its POC administration status: a group of patients that received NAC or AC and a subset of patients that did not receive any chemotherapy. Clinical, oncological and pathological findings were collected and compared between the groups, including age, mFI and CCI median scores, eGFR, cTNM, pTNM, and NAC and AC administration rates.

### 2.4. Study Outcomes

The primary outcome of this study was overall survival (OS). The secondary outcome was the 30-day postoperative complications rate graded by the Clavien–Dindo (CD) classification system according to which minor complications were graded as 1–2, major complications as 3–4, and death as 5 [24]. We specifically evaluated the association between perioperative chemotherapy to OS in the F-M cohort and assessed whether NAC was associated with a higher rate of postoperative complications in this group of patients. 

### 2.5. Statistical Analysis

Statistical comparisons were performed with the Fisher Exact test for categorical variables, and the Mann–Whitney U test for continuous variables. OS was estimated by the Kaplan–Meier method, and the log rank test was applied to compare between groups. Survival was calculated from time of RC until death, and patients who were alive at last follow-up were censored. Cox proportional hazard analyses were generated to calculate hazard ratios (HRs) together with 95% confidence intervals (CIs). We performed multivariable analyses to evaluate the association between POC and OS in the F-M cohort, including either known preoperative predictors for survival (age, gender, eGFR, clinical T stage, clinical N stage) and/or variables that were found to be different in F-M patients. All analyses were two-sided, and statistical significance was defined as *p* < 0.05. SPSS v. 23 (IBM Corp., Armonk, NY, USA) was used to conduct statistical calculations.

## 3. Results

### 3.1. Baseline Characteristics

The study population consisted of 291 patients who underwent RC for non-metastatic MIBC. The median age was 69 years (IQR 63–75) with a male-to-female ratio of 3:1. The median eGFR at presentation was 76 mL/min/1.73 m^2^ (IQR 55–94). The median CCI and mFI scores were 4 (IQR 3–6) and 1 (IQR 0–2), respectively. Fifty-three patients (18%) had a CCI score ≤ 3, and 80 patients (27%) had an mFI score ≤ 1. Serum albumin levels were available for 239 patients, with a median of 40 g/dL (IQR 36–43). POC was administered to 143 patients (49%), with 108 (37%) receiving NAC and 52 (18%) receiving AC. 

Our frailty criteria, defined as both a CCI score ≥ 4 and an mFI score ≥ 2, were met by 102 patients constituting the F-M cohort. Their median CCI and mFI scores were 6 (IQR 5–8) and 2 (IQR 2–3), respectively, with a median serum albumin level of 39 g/dL (IQR 36–43). Table 1 summarizes the clinical baseline characteristics of the F-M cohort and compares them to the non-frail, healthier patients. The patients in the F-M cohort were older (median 72 vs. 67 years, *p* < 0.001) and had a lower preoperative eGFR (median 59 vs. 78 mL/min/1.73 m^2^, *p* < 0.001, Table 1). The F-M patients were barely diverted orthotopically (4%), compared to the non-frail, healthier patients (21%, *p* < 0.001).

### 3.2. Perioperative Chemotherapy

POC was administered to 45 patients in the F-M cohort (44%), 32 (31%) in a neoadjuvant setting, 19 (19%) in an adjuvant setting and 6 (6%) received both. The POC protocols are presented in the Supplementary Table S3. There were no significant differences in the POC administration rates between the groups (*p* = 0.39). Several differences emerged when comparing the F-M patients who received POC to those who did not (Table 2). Those receiving POC were younger (median 74 vs. 72 years, *p* = 0.004), more likely to be females (2.5:1 vs. 10:1, *p* = 0.01) and had a higher preoperative eGFR (median 66 vs. 58 mL/min/1.73 m^2^, *p* = 0.04) and a lower CCI score (median 5 vs. 7, *p* < 0.001) (Table 2). The mFI and albumin levels did not differ between the groups (*p* = 0.27 and *p* = 0.54, respectively). POC in the F-M cohort was not associated with a different likelihood of locally advanced disease (cT3-4 29% vs. 18%, *p* = 0.08) or lymph node involvement (cN+ 16% vs. 7%, *p* = 0.2). There was also no difference between the groups in the orthotopic diversion rates (4% vs. 7%, *p* = 1).

### 3.3. Survival Analyses

**Figure 1:**
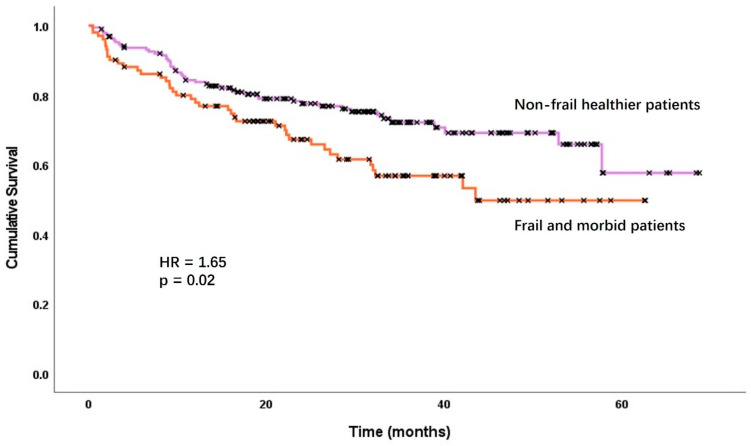
Overall survival stratified by frailty and morbidity category. HR = hazard ratio.

A total of 102 patients died over a median follow-up period of 32 months for survivors (IQR 20–43). The median OS in the F-M cohort was 42 months (95% CI 21–63) and was not reached in the non-frail healthier patients (*p* = 0.02, Figure 1). The estimated 1-, 2- and 3-year OS rates were 77%, 65% and 52% vs. 84%, 77% and 71%, respectively. 

**Figure 2:**
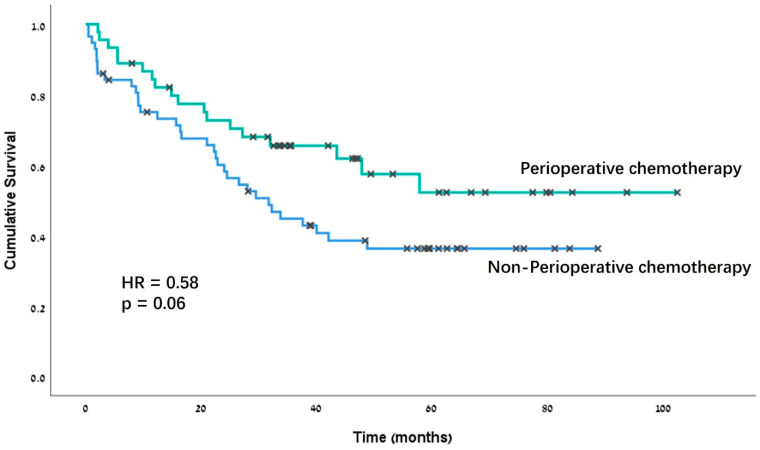
Kaplan–Meier curves for overall survival in the frail and morbid cohort, stratified by perioperative chemotherapy administration. HR = hazard ratio.

In the F-M cohort, the patients who received POC had a higher OS, although this difference did not reach statistical significance (*p* = 0.06, Figure 2). In the univariate Cox regression analysis, POC was found to be a borderline statistically significant predictor of the OS (HR = 0.58, 95% CI 0.33–1.03, *p* = 0.06). However, in a multivariate analysis adjusting for age, eGFR, gender, CCI, and clinical stage, POC was not an independent predictor of survival (Table 3). In a univariate analysis segregating the OS rate according to the timing of POC among the F-M patients, we found that systemic therapy administered in a neoadjuvant setting was associated with improved survival (*p* = 0.004), whereas therapy given in the adjuvant setting did not impact the survival rate (*p* = 0.42). However, in a multivariable analysis adjusting for age, eGFR, gender, CCI and clinical stage, the administration of NAC was not associated with improved OS (HR = 0.5, 95% CI 0.22–1.14, *p* = 1.00). 

### 3.4. Postoperative Complications

The overall and major postoperative complications rates for the study population (n = 291) were 60% and 19%, respectively. In the F-M cohort, 58 patients (57%) experienced postoperative complications, with major complications occurring in 15 patients (15%). There were no significant differences in the overall and major postoperative complication rates between the F-M patients and the non-frail, healthier patients (57% vs. 62%, *p* = 0.53 and 15% vs. 20%, *p* = 0.43, respectively). Moreover, no significant differences were observed in the postoperative complication rates among the F-M patients stratified by the administration of NAC (Table 4).

## 4. Discussion

The management of MIBC in F-M patients represents a significant challenge. While the advantage of combining systemic therapy with bladder removal is unequivocal, its true clinical impact in the F-M subset remains questionable. NAC has been shown to improve the long-term OS by up to 10% in randomized control trials and is considered the standard of care [2,3], while AC has also been shown to improve the OS by up to 10%, but with an inferior level of evidence [4,5,6]. A recent study based on the EORTC30994 with almost 2500 participants found that patients receiving AC displayed a 29% lower probability of dying within 26 months [5]. Within this context, we endeavored to investigate the effects of POC on the OS in an F-M population undergoing RC. Expectedly, we found that F-M patients receiving POC are carefully selected according to their baseline and pathological characteristics. Controlling for established clinical and pathological predictors of survival (age, gender, eGFR, CCI, cT, cN), POC was not associated with a statistically significant OS improvement in this specific F-M population, neither in the neoadjuvant nor in the adjuvant settings.

F-M patients (CCI score ≥ 4 and mFI score ≥ 2) constitute a unique cohort of older, frailer, comorbid patients with lower renal function. Based on evidence that higher scores predict worse outcomes, we selected the mFI and CCI to assess the frailty and comorbidity among RC patients [21,25,26]. CCI is a well-known tool to predict the 5- and 10-year overall mortality, and CCI ≥ 4 is associated with higher rates of rehospitalization, cancer recurrence and 90-day mortality [19,21]. Furthermore, a CCI score of 6 predicts the 5- and 10-year survival rates of 10–20% and 2%, respectively [18]. mFI scores of 2 and above are associated with a worse OS among bladder cancer patients [26]. The survival rates of the F-M patients observed in our study corroborate prior findings, but also imply that the perceived benefit of systemic therapy in MIBC might not provide additional benefit in this unique clinical scenario. 

Despite being frailer and more morbid, the POC administration rate in the F-M cohort was 44%, not different from the rate in the entire study population. In a study investigating octogenarians who underwent RC for MIBC, the perioperative chemotherapy administration rate was 13%, significantly lower compared to younger patients, mostly due to the very low rates of AC utilization [13]. Among frail and sarcopenic patients, the NAC administration rates were not different to their counterparts undergoing RC, ranging between 10 and 35% [17,26,27]. The increased use of POC in vulnerable patients found in our study reflects the ongoing endorsement of clinical guidelines highlighting the cardinal role of a multimodal treatment approach in optimizing clinical outcomes. Yet, the lack of an associated OS advantage may raise several concerns. 

The inherent lower OS of F-M patients undergoing RC for MIBC (52% @ 3 years) might obscure the potential benefit of POC on long-term survival. Moreover, limited physiological reserves and the considerable toxicity associated with Cisplatin-based chemotherapy in F-M patients could offset the potential benefits of therapy and diminish its therapeutic advantage. Other factors, such as age and clinical T staging, might play a more substantial role in determining survival outcomes among these patients. With the observed increase in life expectancy and increased number of F-M patients who will be diagnosed with MIBC, our findings question the general application of standard POC regimens to frail and/or morbid population and contribute to the ongoing discourse on optimizing treatment strategies in these patients.

Our study’s retrospective design introduces inherent limitations, such as the potential selection bias and reliance on the accuracy of recorded data. Secondly, the retrospective assessment of frailty and comorbidities using indices such as the CCI and mFI may not fully capture the dynamic clinical status of patients over time. Thirdly, the sample size, while substantial, may still be insufficient to detect smaller but clinically significant differences in outcomes. Fourth, we lacked data regarding the cause of death due to an inability to categorize it appropriately. Fifth, we lacked data regarding the smoking status. Finally, data regarding POC toxicity and adverse events were lacking, withholding an additional important layer when deliberating chemotherapy for F-M patients. 

## 5. Conclusions

Our retrospective multicenter study found that the administration of POC was not associated with improved OS in frail and morbid patients undergoing RC for MIBC. The CCI and mFI may serve as clinical tools for identifying frail and morbid patients. These findings highlight the need for personalized treatment strategies and further prospective studies to optimize care in this unique and growing population.

## Figures and Tables

**Table 1:** Clinical baseline characteristics of F-M and non-frail patients.

Variables	F-M Cohort (n = 102)	Non-Frail, Healthier Patients (n = 189)	*p*-Value
Age at surgery [years], median (IQR)	72 (IQR 68–79)	67 (61–73)	**<0.001**
eGFR at presentation [mL/min/1.73 m^2^], median (IQR)	59 (IQR 44–87)	78 (59–96)	**<0.001**
Albumin [g/dL], median (IQR)	39 (36–43)	40 (36–43)	0.58
CCI score, median (IQR)	6 (5–8)	3 (2–5)	**<0.001**
mFI score, median (IQR)	2 (2–3)	1 (0–1)	**<0.001**
Orthotopic diversion, n (%)	4 (4%)	39 (21%)	**<0.001**
Perioperative chemotherapy, n (%)	45 (44%)	97 (51%)	0.39
NAC, n (%)	32 (31%)	76 (40%)	0.20
AC, n (%) Both, n (%)	19 (19%) 6 (6%)	33 (17%) 12 (6%)	0.75 1.00

AC = adjuvant chemotherapy; CCI = Charlson Comorbidity Index; eGFR = estimated glomerular filtration rate; F-M = frail and morbid; IQR = Interquartile Range; mFI = Modified Frailty Index; NAC = neoadjuvant chemotherapy. **Bold** indicates significance.

**Table 2:** Clinical, oncological and pathological characteristics of F-M cohort (n = 102), stratified by perioperative chemotherapy administration.

Variables	Non-POC (n = 57)	POC (n = 45)	*p*-Value
Clinical variables			
Age at surgery [years], median (IQR)	74 (69–81)	72 (66–76)	**0.004**
eGFR at presentation [mL/min/1.73 m^2^], median (IQR)	58 (36–86)	66 (48–99)	**0.04**
Male-to-female ratio	10:1	2.5:1	**0.01**
CCI score, median (IQR)	7 (5–8)	5 (4–6)	**<0.001**
mFI score, median (IQR)	2 (2–3)	2 (2–3)	0.27
Albumin [g/dL], median (IQR) Orthotopic diversion, n (%)	39 (36–43) 2 (4%)	39 (36–43) 2 (7%)	0.54 1.00
Preoperative oncological variables			
Clinical T stage			0.08
cT1-2, n (%)	49 (86%)	32 (71%)	
cT3-4, n (%)	8 (14%)	13 (29%)	
Clinical N stage			0.2
cN0, n (%)	53 (93%)	38 (84%)	
cN+, n (%)	4 (7%)	7 (16%)	
Pathological variables			
Pathological T stage			**0.01**
pT0, n (%)	7 (12%)	11 (24%)	
pT1-2, n (%)	25 (44%)	8 (18%)	
pT3-4, n (%)	25 (44%)	26 (58%)	
Pathological N stage			**0.02**
pN0, n (%)	46 (80%)	26 (58%)	
pN+, n (%)	11 (20%)	19 (42%)	
Prostate cancer, n (%) Grade 1 Grade 2+	6 (11%) 5 (9%) 1 (2%)	8 (18%) 5 (11%) 3 (7%)	0.39

CCI = Charlson Comorbidity Index; eGFR = estimated glomerular filtration rate; F-M = frail and morbid; IQR = Interquartile Range; mFI = Modified Frailty Index; POC = perioperative chemotherapy. **Bold** indicates significance.

**Table 3:** Multivariate Cox regression model for overall survival of F-M patients.

Variables	HR	95% CI	*p*-Value
Perioperative chemotherapy	0.79	0.4–1.56	0.50
Age	1.08	1.03–1.14	**0.002**
eGFR	0.99	0.98–1.01	0.59
Gender	2.99	1.06–8.42	**0.03**
CCI	1.06	0.92–1.21	0.40
Clinical T staging	1.57	1.08–2.27	**0.01**
Clinical N staging	1.72	0.89–3.3	0.10

CCI = Charlson Comorbidity Index; CI = confidence interval; eGFR = estimated glomerular filtration rate; F-M = frail and morbid; HR = hazard ratio. **Bold** indicates significance.

**Table 4:** Postoperative complications rates in the F-M cohort, stratified by NAC administration.

Variables	NAC (n = 32)	Non-NAC (n = 70)	*p*-Value
Overall complications, n (%)	15 (47%)	47 (67%)	0.20
Major complications, n (%)	2 (6%)	13 (19%)	0.14
Minor complications, n (%)	13 (41%)	34 (48%)	0.52

F-M = frail and morbid; NAC = neoadjuvant chemotherapy.

## Data Availability

The data presented in this study are available on request from the corresponding author.

## Supplementary Materials

The following supporting information can be downloaded at https://www.mdpi.com/article/10.3390/jpm14090954/s1, Table S1: Modified Frailty Index; Table S2: Charlson Comorbidity Index; Table S3: Perioperative Chemotherapy Protocols.
