# Supplementary material for: Evaluating the Survival Benefits of Perioperative Chemotherapy in Frail and Morbid Muscle-Invasive Bladder Cancer Patients
Source: J Pers Med. 2024 Sep 9;14(9):954. doi: 10.3390/jpm14090954 (PMC11433015; doi:10.3390/jpm14090954)
Supplement: Supplementary file 1 [file jpm-14-00954-s001.zip › jpm-3175961-supplementary.pdf]

Supplementary Table S1: Modified Frailty Index

Comorbidities	*Score*
Functional health status before surgery: partially or totally dependent	*1*
Diabetes mellitus type II	*1*
Chronic Obstructive Pulmonary Disease	*1*
Congestive heart failure	*1*
History of myocardial infarction within past 6 months	*1*
Prior cardiac surgery, percutaneous coronary intervention, or angina within past month	*1*
Hypertension	*1*
Impaired sensorium	*1*
Peripheral vascular disease requiring surgery or active claudication present	*1*
History of transient ischemic attack	*1*
History of cerebrovascular accident	*1*

Supplementary Table S2: Charlson Comorbidity Index

Comorbidities	*Score*
Myocardial infraction	*1*
Congestive heart failure	*1*
Peripheral Vascular Disease	*1*
Cerebrovascular Disease	*1*
Dementia	*1*
Chronic Obstructive Pulmonary Disease	*1*
Connective Tissue Disease	*1*
Ulcer Disease	*1*
Mild Liver Disease	*1*
Diabetes	*1*
Hemiplegia	*2*
Moderate or Severe Renal Disease	*2*
Diabetes with End-Organ Damage	*2*
Any tumor	*2*
Leukemia	*2*
Lymphoma	*2*
Moderate or Severe Liver Disease	*3*
Solid Tumor with Metastasis	*6*
Acquired Immunodeficiency Syndrome	*6*
Each decade over 40 years	*1*

Supplementary Table S3 . Perioperative chemotherapy protocols.

Regimen	Cycles
MVAC – methotrexate, vinblastine, doxorubicin, cisplatin	4-6
Cisplatin-Gemcitabine	4-6
Carboplatin-Gemcitabine	4
